# Supplementary material for: 11β-HSD1 Inhibitor Alleviates Non-Alcoholic Fatty Liver Disease by Activating the AMPK/SIRT1 Signaling Pathway
Source: Nutrients. 2022 Jun 6;14(11):2358. doi: 10.3390/nu14112358 (PMC9182913; doi:10.3390/nu14112358)
Supplement: Supplementary file 1 [file nutrients-14-02358-s001.zip › nutrients-1734081-supplementary.pdf]

nutrients

MDPI

*Article*

# 11β-HSD1 Inhibitor Alleviates Non-Alcoholic Fatty Liver Disease by Activating the AMPK/SIRT1 Signaling Pathway

**Ying Chen, Jiali Li, Meng Zhang, Wei Yang, Wenqi Qin, Qinzhou Zheng, Yanhui Chu, Yan Wu, Dan Wu and Xiaohuan Yuan ***

Heilongjiang Key Laboratory of Tissue Damage and Repair, College of Life Science, Mudanjiang Medical University, Mudanjiang 157011, China; chenying9435@126.com (Y.C.); lijiali0530@163.com (J.L.); zhangmengzi1125@163.com (M.Z.); yangwei19970531@163.com (W.Y.); qinwenqi0717@163.com (W.Q.); zz804873737@163.com (Q.Z.); yanhui_chu@sina.com (Y.C.); wuyan@mdjmu.edu.cn (Y.W.); wudan@mdjmu.edu.cn (D.W.)

* Correspondence: yuanxiaohuan@mdjmu.edu.cn; Tel.: +86-0453-6984401

**Abstract:** We investigated the effect of an 11β-HSD1 inhibitor (H8) on hepatic steatosis and its mechanism of action. Although H8, a curcumin derivative, has been shown to alleviate insulin resistance, its effect on non-alcoholic fatty liver disease (NAFLD) remains unknown. Rats were fed a high-fat diet (HFD) for 8 weeks, intraperitoneally injected with streptozotocin (STZ) to induce NAFLD, and, then, treated with H8 (3 or 6 mg/kg/day) or curcumin (6 mg/kg/day) for 4 weeks, to evaluate the effects of H8 on NAFLD. H8 significantly alleviated HFD+STZ-induced lipid accumulation, fibrosis, and inflammation as well as improved liver function. Moreover, 11β-HSD1 overexpression was established by transfecting animals and HepG2 cells with lentivirus, carrying the 11β-HSD1 gene, to confirm that H8 improved NAFLD, by reducing 11β-HSD1. An AMP-activated protein kinase (AMPK) inhibitor (Compound C, 10 μM for 2 h) was used to confirm that H8 increased AMPK, by inhibiting 11β-HSD1, thereby restoring lipid metabolic homeostasis. A silencing-related enzyme 1 (SIRT1) inhibitor (EX572, 10 μM for 4 h) and a SIRT1 activator (SRT1720, 1 μM for 4 h) were used to confirm that H8 exerted anti-inflammatory effects, by elevating SIRT1 expression. Our findings demonstrate that H8 alleviates hepatic steatosis by inhibiting 11β-HSD1, which activating the AMPK/SIRT1 signaling pathway.

**Keywords:** 11-beta-hydroxysteroid dehydrogenase type 1 (11β-HSD1); curcumin; non-alcoholic fatty liver disease (NAFLD); lipid metabolism; anti-inflammatory

**Citation:** Chen, Y.; Li, J.; Zhang, M; Yang, W; Qin, W; Zheng, Q; Chu, Y; Wu, D; Wu, Y; Yuan, X. 11β-HSD1 Inhibitor Alleviates Non-Alcoholic Fatty Liver Disease by Activating the AMPK/SIRT1 Signaling Pathway. *Nutrients* **2022**, *14*, 2358. https://doi.org/10.3390/nu14112358

Academic Editor: Antoni Sureda

Received: 4 May 2022
Accepted: 5 June 2022
Published: 6 June 2022

**Publisher's Note:** MDPI stays neutral with regard to jurisdictional claims in published maps and institutional affiliations.

**Copyright:** © 2022 by the authors. Submitted for possible open access publication under the terms and conditions of the Creative Commons Attribution (CC BY) license (https://creativecommons.org/licenses/by/4.0/).

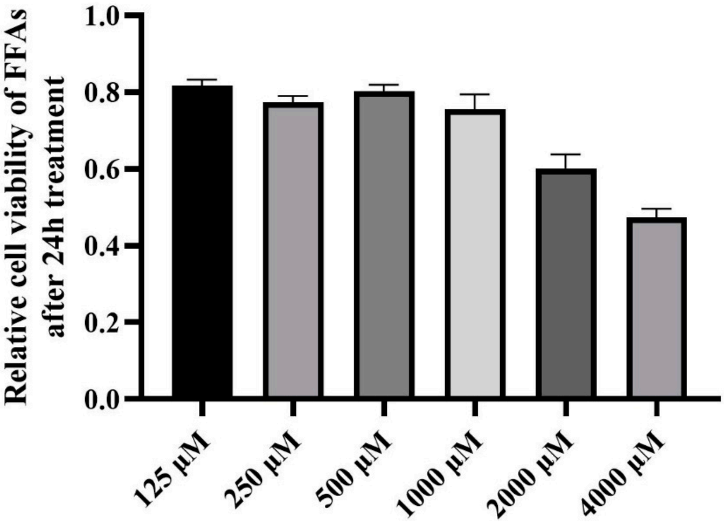


**Supplementary Figure S1.** Relative cell viability of FFAs after 24h treatment.

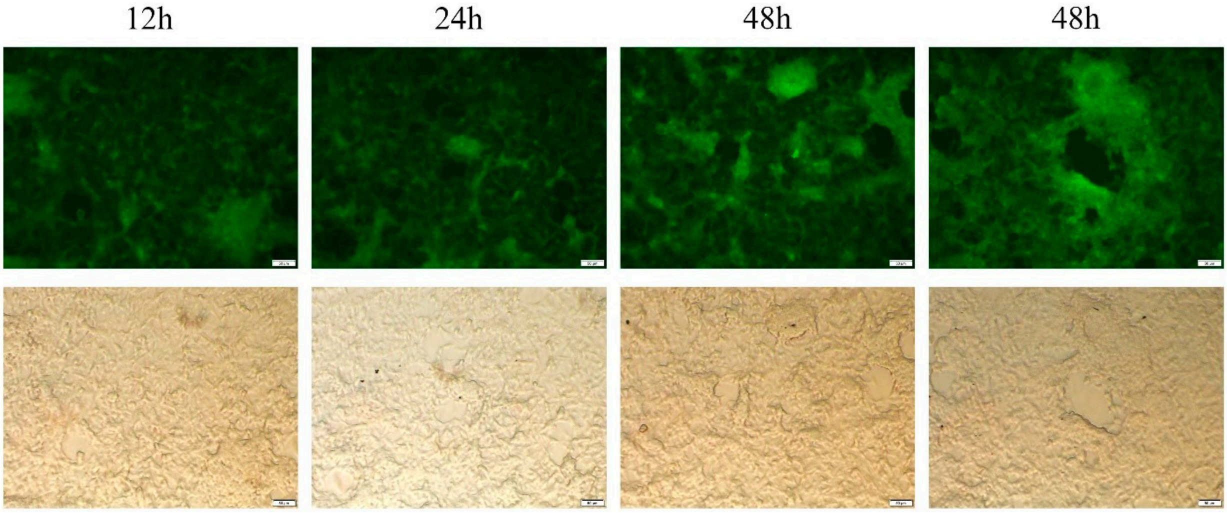


**Supplementary Figure S2.** Transfection efficiency

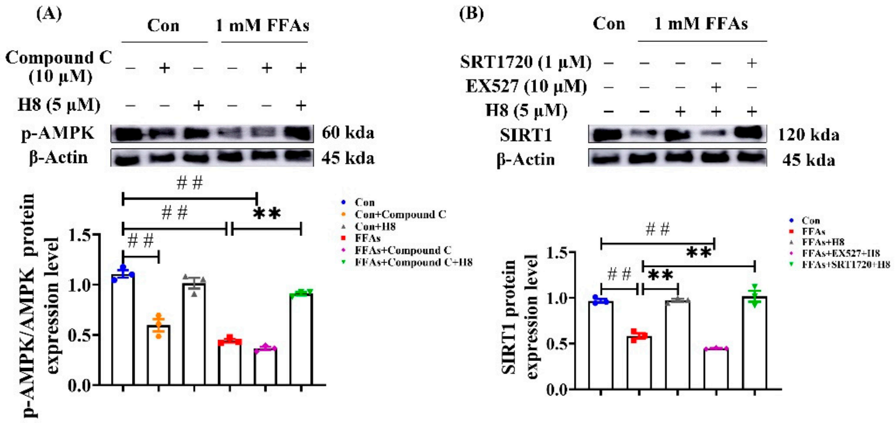


**Supplementary Figure S3.** AMPK and SIRT1 protein expression levels in HepG2 cells. $n = 8$, # $p < 0.05$, ## $p < 0.01$ versus the control; * $p < 0.05$, ** $p < 0.01$ versus the HFD+STZ; ns $p > 0.05$ not significant.

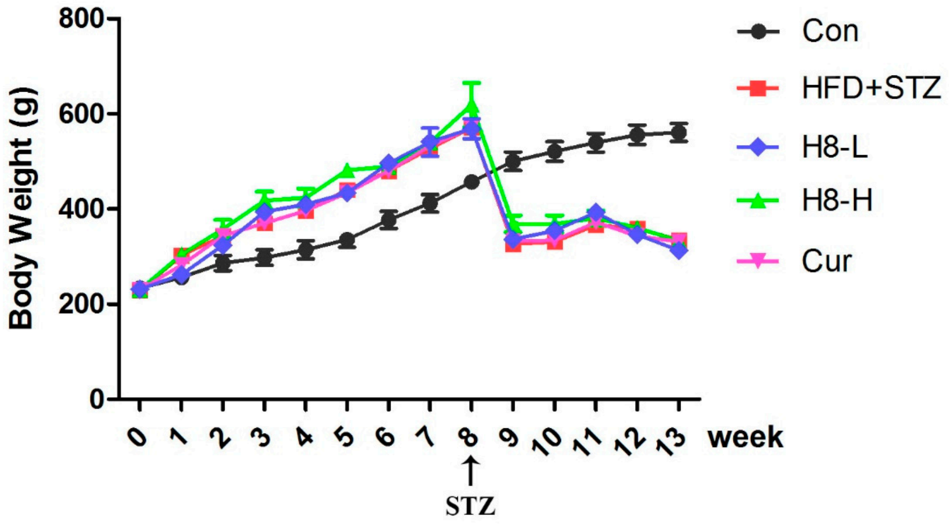


**Supplementary Figure S4**. Body weight change

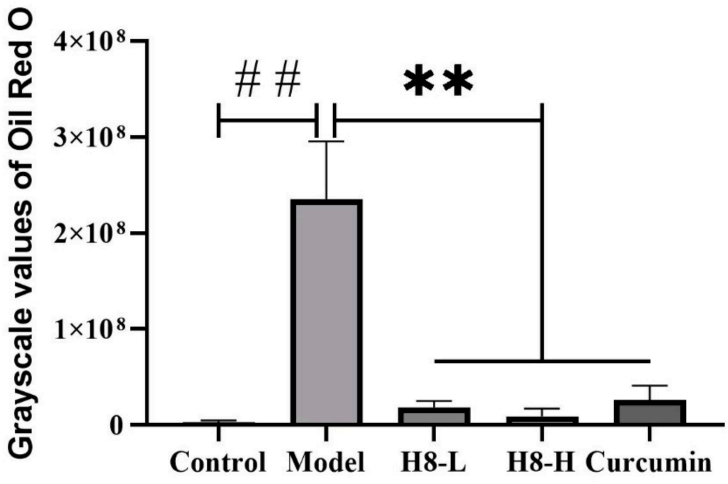


**Supplementary Figure S5.** Grayscale values of Oil Red O in rat liver. $n = 8$, # $p < 0.05$, ## $p < 0.01$ versus the control; * $p < 0.05$, ** $p < 0.01$ versus the HFD+STZ; ns $p > 0.05$ not significant.

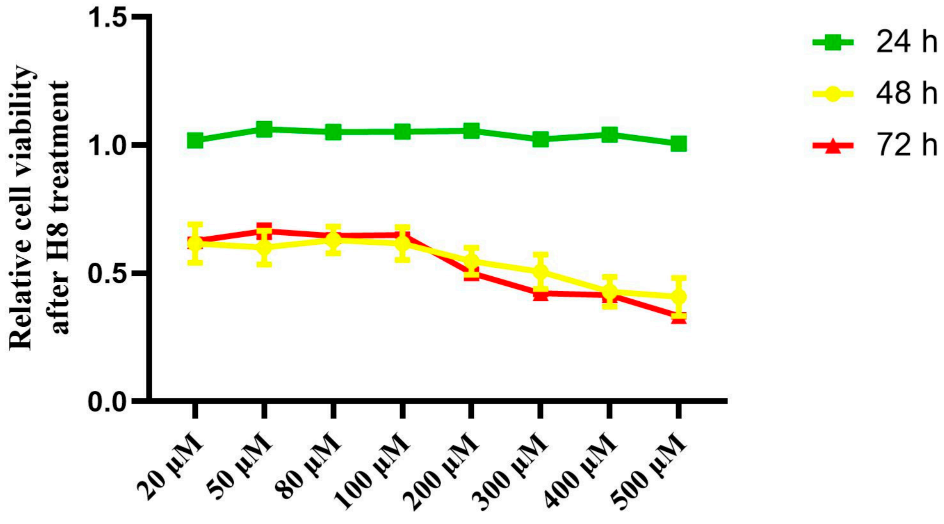


**Supplementary Figure S6.** Relative cell viability after H8 treatment.

**Supplementary Table S1.** The primer sequences used in qPCR.

| Gene | Forward | Reverse |
|---|---|---|
| GAPDH (rat) | TGTGAAGCTCATTTCCTGGTAT | GATGGGGACTCCTCAGCAAC |
| GAPDH (human) | CACCATCTTCCAGGAGCGAG | TGATGACCCTTTTGGCTCCC |
| β-Actin (mouse) | CCTAGGCACCAGGGTGTGAT | AGCACAGGGTGCTCCTCA |
| SIRT1 (mouse) | TCGGCTACCGAGGTCCATA | CCGCAAGGCGAGCATAGATA |
| SIRT1 (rat) | TGGAAGGAAAGCAATTTTGGAAATA | CTGCAACCTGCTCCAAGGTA |
| SIRT1 (human) | CATTCTTCAAGTTTGCAAAGGAAAT | CGAAGTAGTTTCCTTCCTTATCTG |
| HSD1-11β (mouse) | ACTCAGACCTCGCTGTCTCT | TGGGTCATTTTCCCAGCCAA |
| HSD1-11β (rat) | CTCCTCCATGGCTGGGAAAA | GAAGCCGAGGACACAGAGAG |
| SREBP1 (rat) | TCTTGACCGACATCGAAGACAT | GCCTGTGTCTCCTGTCTCAC |
| SREBP1 (human) | CTGACCGACATCGAAGGTGA | CCAGCATAGGGTGGGTCAAA |
| HSL (rat) | GTCAAACCCTCCAGAGCCAA | GTGAGAATGCCGAGGCTGTA |
| HSL (human) | CCTCGTCCTCACTCCTCCC | TTAAGTAAGGCACAGCCCGC |
| FAS (rat) | TGTACCCTCTAGCTGGACCC | CCAGGCTAAGGGCAATGGAA |
| FAS (human) | TCGTGTTGACTTCTCGCTCC | CCATCTCTCAAGACCACGGC |
| TNF-α (rat) | ATGGGCTCCCTCTCATCAGT | GCTTGGTGGTTTGCTACGAC |
| TNF-α (human) | TCTCCTTCCTGATCGTGGCA | CAGCTTGAGGGTTTGCTACAAC |
| PGC-1α (rat) | TGGAGTGACATAGAGTGTGCTG | TATGTTCGCGGGCTCATTGT |
| PGC-1α (human) | TCTGACCCCAGAGTCACCAA | GTGGAGTTAGGCCTGCAGTT |
| PPAR-γ (rat) | GCTTGTGAAGGATGCAAGGG | GCCCAAACCTGATGGCATTG |
| PPAR-γ (human) | GCAATCAAAGTGGAGCCTGC | TCTCCGGAAGAAACCCTTGC |

**Supplementary Table S2.** The primary antibodies used in WB.

| Gene | Forward | Reverse |
|---|---|---|
| β-actin | Cell Signaling Technology | 1:1000 |
| SIRT1 | Affinity | 1:1000 |
| 11β-HSD1 | Abcam | 1:1000 |
| AMPK-α1 | Abcam | 1:1000 |
| p-AMPK-α1 | Abcam | 1:1000 |
| sterol regulatory element binding protein 1 (SREBP1) | Abcam | 1:1000 |
| fatty acid synthase (FASN) | Affinity | 1:1000 |
| carnitine palmitoyltransferase 1β (CPT-1β) | Abcam | 1:1000 |
| acetyl coenzyme A carboxylase (ACC1) | Abcam | 1:1000 |
| p-ACC1 | Cell Signaling Technology | 1:1000 |
| hormone-sensitive triglyceride lipase (HSL) | Abcam | 1:1000 |
| interleukin-6 (IL-6) | Abcam | 1:500 |
| interleukin-17 (IL-17) | Abcam | 1:1000 |
| tumor necrosis factor-α (TNF-α) | Bioss | 1:500 |
| peroxisome proliferator-activator receptor gamma (PPAR-γ) | Affinity | 1:1000 |
| peroxisome proliferator-activated receptor gamma coactivator 1-α (PGC-1α) | Affinity | 1:1000 |
| nuclear factor kappa-B p65 (NF-kB p65) | Abcam | 1:1000 |
| NF-κB-p-p65 | Affinity | 1:1000 |
